# Functional hydrogel coatings

**DOI:** 10.1093/nsr/nwaa254

**Published:** 2020-10-05

**Authors:** Junjie Liu, Shaoxing Qu, Zhigang Suo, Wei Yang

**Affiliations:** Center for X-Mechanics, Key Laboratory of Soft Machines and Smart Devices of Zhejiang Province and Department of Engineering Mechanics, Zhejiang University, Hangzhou 310027, China; State Key Laboratory of Fluid Power and Mechatronic System, Zhejiang University, Hangzhou 310027, China; Applied Mechanics and Structure Safety Key Laboratory of Sichuan Province, School of Mechanics and Engineering, Southwest Jiaotong University, Chengdu 610031, China; Center for X-Mechanics, Key Laboratory of Soft Machines and Smart Devices of Zhejiang Province and Department of Engineering Mechanics, Zhejiang University, Hangzhou 310027, China; State Key Laboratory of Fluid Power and Mechatronic System, Zhejiang University, Hangzhou 310027, China; John A. Paulson School of Engineering and Applied Sciences, Kavli Institute for Bionano Science and Technology, Harvard University, Cambridge, MA 02138, USA; Center for X-Mechanics, Key Laboratory of Soft Machines and Smart Devices of Zhejiang Province and Department of Engineering Mechanics, Zhejiang University, Hangzhou 310027, China

**Keywords:** hydrogel coatings, coating methods, coating tests, adhesion, hydrogel applications

## Abstract

Hydrogels—natural or synthetic polymer networks that swell in water—can be made mechanically, chemically and electrically compatible with living tissues. There has been intense research and development of hydrogels for medical applications since the invention of hydrogel contact lenses in 1960. More recently, functional hydrogel coatings with controlled thickness and tough adhesion have been achieved on various substrates. Hydrogel-coated substrates combine the advantages of hydrogels, such as lubricity, biocompatibility and anti-biofouling properties, with the advantages of substrates, such as stiffness, toughness and strength. In this review, we focus on three aspects of functional hydrogel coatings: (i) applications and functions enabled by hydrogel coatings, (ii) methods of coating various substrates with different functional hydrogels with tough adhesion, and (iii) tests to evaluate the adhesion between functional hydrogel coatings and substrates. Conclusions and outlook are given at the end of this review.

## Teaser

The emerging topic of functional hydrogel coatings are reviewed from three aspects: functions and applications of hydrogel coatings, methods of preparing hydrogel coatings with strong adhesion, and tests to evaluate the adhesion.

## INTRODUCTION

Hydrogels, a family of soft matter [1], are aggregates of water molecules and hydrophilic polymer networks. The high water content, which can exceed 95% by weight ratio, enables hydrogels to dissolve and transport ions and many small molecules. The polymer networks, which are often sparsely crosslinked, lead to hydrogels being soft and elastic. Hydrogels may have originated with the beginning of life on earth. They are ubiquitous in nature, from muscle and cartilage in animal tissues to xylems and phloems in plants [2–4]. The first report on synthetic hydrogels used in biomedicine was published in 1960 in literature [5]. Since then, the development of hydrogels with new applications and enhanced mechanical robustness has attracted researchers across chemistry, physics, engineering and medicine.

**Figure 1.**
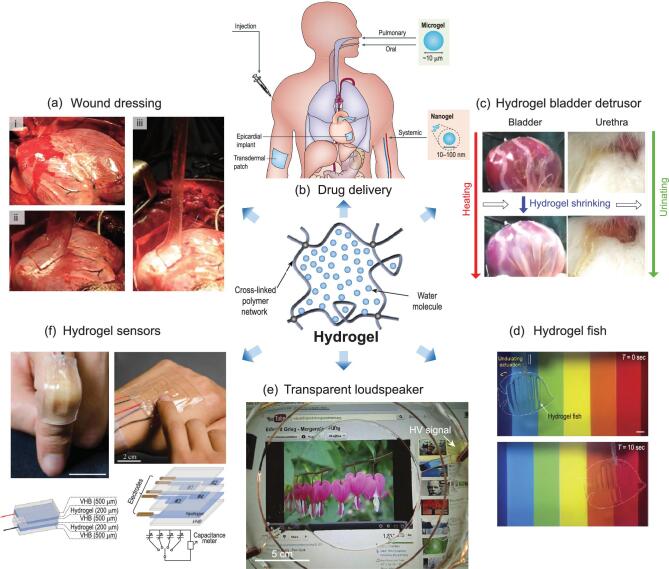
Typical applications of hydrogels. (a) A hydrogel seals a cut on a pig heart with strong adhesion. The figure is redrawn from ref [9]. Reprinted with permission from AAAS (American Association for the Advancement of Science). (b) Hydrogels in drug delivery applications. The feature size of the drug-loaded hydrogel dictates the pertinent delivery routes. The figure is redrawn with permission from ref [12], Springer Nature. (c) A hydrogel actuator, used as a bladder detrusor, can help to shrink an animal bladder. The figure is adapted with permission from ref [14], John Wiley and Sons. (d) An optically and sonically camouflaged hydraulic hydrogel fish in water. The figure is adapted with permission from ref [16], Springer Nature. (e) A novel transparent loudspeaker made from ionically conductive hydrogel and dielectric elastomer. The figure is adapted from ref [33]. Reprinted with permission from AAAS. (f) Hydrogel sensors for stretch and compression testing. The figure is adapted with permission from ref [20], John Wiley and Sons.

In industrial applications, hydrogels are relatively new compared with metals, ceramics and many other forms of polymer. The diversity of hydrogels, natural and synthetic, with different polymer topologies and chemical compositions, makes them highly adaptive to a vast array of applications [6]. Functional hydrogels can chemically, mechanically and electrically mimic the functions of biological tissues [4]. Established medical applications of functional hydrogels include tissue engineering [7], wound dressing (Fig. 1a) [8,9], contact lenses [10] and drug delivery (Fig. 1b) [11,12]. Hydrogels also play key roles in stretchable devices and soft robotics, such as muscle-like actuators (Fig. 1c) [13–15], hydrogel fish (Fig. 1d) [16], soft displays [17], stretchable ionotronics (Fig. 1e) [18,19], skin-like sensors (Fig. 1f) [20] and axon-like interconnects [21,22].

One critical challenge in the application of hydrogels is achieving strong bonding between hydrogels and other materials. For instance, hydrogels have long been developed as adhesives for wound closure, however for decades the adhesion was limited below the order of 10 J/m^2^ [23]. A transformative advance took place when Yuk *et al.* adhered hydrogels to non-porous surfaces [24] and elastomers [25] with adhesion greater than 1000 J/m^2^. The high adhesion was achieved through the synergy of two effects: the covalent bonds between the polymer network in the hydrogel and the substrate, and the energy-dissipating sacrificial bonds in the hydrogel. Since the publication of these studies, other strategies for achieving tough hydrogel adhesion have been reported, such as stitching wet materials (tissues and hydrogels) with biocompatible polymer chains [26], and *insitu* bonding of dissimilar polymer networks by bulk modification using silanes [27]. Hydrogel adhesion is a supramolecular synergy of chemistry, topology and mechanics [28].

Functional hydrogels have been successfully coated onto various substrates of arbitrary shapes with strong bonds [24,25,29–32]. The hydrogel-coated substrates combine the functions of both the substrates and hydrogels, enabling new functions and applications, for example as biocompatible medical devices [31], lubricious nitinol guidewire in medicine [32] and a transparent loudspeaker [33]. In addition, hydrogel coatings play an essential role as structural components in the emerging field of

hydrogel machines, which focuses on devices and robotics that incorporate hydrogels. Liu *et al.* reviewed recent advances in hydrogel machines [34], describing the functions enabled by hydrogel coatings and various interactions between hydrogel coatings and substrates.

This paper reviews the emerging topic of functional hydrogel coatings. Emphasis is placed on their functions and applications, fabrication, and methods to evaluate their adhesion. We list the functions and potential applications of hydrogel coatings reported in literatures. Methods that allow various hydrogels to be coated onto different types of substrate with arbitrary shapes are addressed. Finally, we highlight the thickness-dependent adhesion of hydrogel coatings and the experimental methods for evaluating the adhesion. Conclusions and outlook are given at the end of this review.

## FUNCTIONS AND APPLICATIONS OF HYDROGEL COATINGS

Hydrogel coatings endow the coated bulk materials with new functions, while having negligible influence on the mechanical properties of the bulk materials. The diversity of hydrogels in chemical components and network topologies makes them appealing candidates as coating materials. The functions and applications enabled by hydrogel coatings can be roughly classified as medical (Table 1) and non-medical applications (Table 2). The details of these applications will be discussed in the sequel.

**Table 1.** Hydrogel coatings in medical area.

Function/property	Hydrogel	Substrate/device	Bonding	Drug/nanoparticle	Reference

Drug delivery	Hyaluronan and poly-D, L-Lactide	cobalt-chrome, polyethylene, titanium	PA	vancomycin, amikacin, tobramycin, gentamicin, sodium salicylate	[35]
	Gelatin	titanium alloy	PA	antimicrobial peptide, laponite nanosilicate	[36]
	PEG-heparin^a^	polyurethane	CA	silver nitrate	[37]
	PMBV/PVA	titanium alloy	CA	paclitaxel (PTX)	[38]
	PHEMA	titanium alloy	PA	ciprofloxacin (CIP)	[39]
	PEGPLA	iridium oxide	PA	nerve growth factor (NGF)	[40]
	PEGDA-co-AA	titanium	PA	silver nanoparticle	[41]
	PEGDA	stainless steel	PA	copper nanoparticles	[42]
	PHEMA	silicone	CA	nerve growth factor (NGF)	[43]
Lubricity	Chitosan/PVA	polyurethane urethral catheter	CA		[44]
	PAAm	nitinol guidewire	CA		[32]
	Chitosan	polyethylene tube	CA		[45]
	Chitosan	endovascular catheter	CA		[46]
Anti-biofouling	PEG	polyamide composite	CA		[47]
	PEG/polycarbonate	silicone rubber	CA		[48]
	Zwitterionic PCB	powdered carbon	PA		[49]
	HEMA-co-DHPMA	glucose sensor	PA		[50]
	pCBAA	gold/silicon dioxide	PA		[51]
	PEGMA	gold	CA		[52]
Conductive	PVA/PAA	iridium oxide	CA		[53,54]
coatings for	PU	iridium oxide	CA		[55]
neural electrode	Alginate/PPy	gold	PA		[56]
	Alginate/PEDOT	gold	PA		[57]
	PVA/PEDOT	platinum	PA		[58]

^a^AA, acrylic acid; CA, covalent anchorage; DHPMA, 2,3-dihydroxypropyl methacrylate; PA, physical attachment; PAA, poly(acrylic acid); PAAm, polyacrylamide; PCB, poly(carboxybetaine); pCBAA, poly(carboxybetaine acrylamide); PEDOT, poly(3,4-ethylenedioxythiophene); PEG, poly(ethylene glycol); PEGDA, poly(ethylene glycol diacrylate); PEGMA, poly(ethylene glycol) methacrylate; PEGPLA, poly(ethylene glycol)-poly(lactic acid); PHEMA, poly(2-hydroxyethyl methacrylate); PMBV, poly(2-methacryloyloxyethyl phosphorylcholine-co-n-butyl methacrylate-co-p-vinylphenylboronic acid); PPy, polypyrrole; PVA, polyvinyl alcohol; PU, polyurethane.

**Table 2.** Hydrogel coatings in non-medical area.

Sensing					
Hydrogel	Substrate	Bonding	Detection	Output	Reference

Poly(HEMA-co-AAc)	silicon wafer	CA	volatile vapor	optical signal	[67]
Poly(AAm-co-AAc)					
poly(AAm-co-AAc-co -AAene) with imidazole ligands or PBA^a^	silicon wafer, glass, PET, PDMS	CA	copper ions, glycoprotein	optical signal	[68]
Gelatin	long-period grating (LPG)	CA	humidity	optical signal	[69]
GPBS imprinted PAH	gold	CA	glucose	SPR spectrum	[70]
PAAm	FBG	PA	salt concentration	transmission spectrum	[71]
Actuation					
Hydrogel	Substrate	Bonding		Stimulation	Reference
PAA	PDMS	CA		pH	[32]
PNIPAM, PAA, PEODA	hydrogel	IP		pH, ionic strength	[72]
PVA-DEEDA-borax gel	BOPP	HB		moisture, temperature, light	[73]
PNIPAM	Glass	CA		temperature	[74]
PAAm	PDMS	CA		humidity	[75]
PNIPAM/PVA	PDMAEMA/PSS	IP		temperature, pH	[76]
Anti-marine creature fouling					
Hydrogel	Substrate	Bonding		Marine creature	Reference
PAMPA/PAAm, PVA	polyethylene	FA		algae, sea squirts, barnacles	[77]
PVA-glycerol	stainless steel	FA		barnacle (balanus albicostatus)	[78]
PEG	stainless steel, nylon	HB		diatom	[79]
PEG	glass, silicon, PS	CA		barnacle, algal zoospores, diatom	[80]
MMA-co-AA-co-TBSM	glass	FA		barnacle	[81]
PEG	glass	CA		marine bacteria and diatom	[82]
Oil-water separation					
Hydrogel	Substrate	Bonding		Contaminant	Reference
DKGM Hydrogel	glass fabric	PA		oil, organic dyes, and heavy metals	[83]
PAAm	magnetic nickel foam	PA		oil, dichloromethane	[84]
PAAm	stainless steel mesh	PA		vegetable oil, gasoline, diesel, and crude oil	[85]
Alginate	PAA-g-PVDF	IB		crude oil	[86]
Alginate	filter paper	PA		crude oil	[87]

^a^BOPP, polypropylene; DEEDA, N, N-diethylethane-1,2-diamine; DKGM, deacetylated konjac glucomannan; GPBS, glucose phosphate barium salt; HB, hydrogen bond; IB, ionic bond; IP, interfacial penetration; NaCl, sodium chloride; PAA, polyacrylic acid; PAAm, polyacrylamide; PAH, poly(allylamine hydrochloride); PBA, phenylboronic acid; PBG, fiber Bragg grating; PDMAEMA, poly(2-(dimethylamino)ethyl methacrylate); PDMS, poly(dimethylsiloxane); PEG, polyethylene glycol; PET, poly(ethylene terephthalate); PNIPAM, poly(N-isopropylacrylamide); PS, polystyrene; PSS, poly(sodium-p-styrenesulfonate); PVA, poly(vinyl alcohol); PVDF, poly(vinylidene fluoride); SPR, surface plasmon resonance.

### Drug delivery

**Figure 2.**
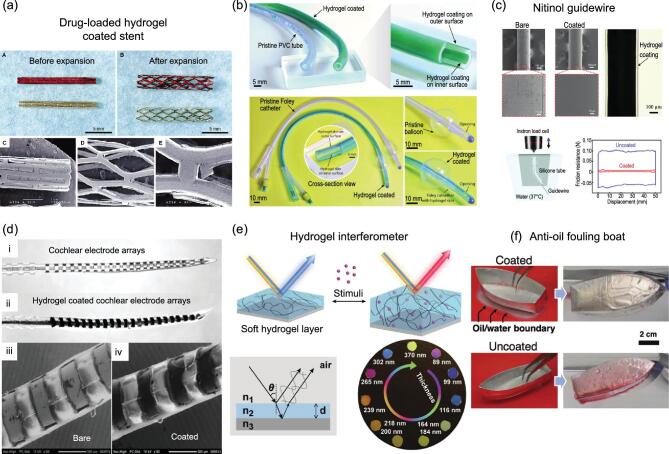
Examples of hydrogels in coating applications. (a) Drug-loaded hydrogel-coated metallic stents. The figure is modified with permission from ref [59], John Wiley and Sons. (b) Slippery hydrogel coating on biomedical tubing and a Foley catheter. The figure is modified with permission from ref [31], John Wiley and Sons. (c) Lubricious hydrogel coating on a nitinol guidewire used in surgical operation. The figure is redrawn with permission from ref [32], John Wiley and Sons. (d) Biocompatible and conductive hydrogel coating on cochlear electrode arrays made from platinum. The figure is adapted with permission from ref [58], IEEE (Institute of Electrical and Electronics Engineers). (e) Stimuli-responsive hydrogel coatings in a sensing system based on the interference of light. The figure is modified with permission from ref [67], John Wiley and Sons. (f) Anti-oil fouling hydrogel coating on a model boat. The figure is redrawn with permission from ref [32], John Wiley and Sons.

Hydrogels are appealing candidates for use as carriers in drug delivery systems that transport drugs to target sites and release them at a controlled rate. This drug delivery, controlled in both space and time, improves the accuracy of drug allocation and results in fewer side effects. The feature size of a hydrogel dictates the possible delivery routes. For example, a drug-loaded transdermal hydrogel patch releases a drug by attaching to the human body and a drug-loaded microgel transports a drug via oral or pulmonary delivery. Details for the design of hydrogels for controlled drug delivery can be found in a recent review [12]. The main functions of drugs or nanoparticles loaded into hydrogel coatings on implanted medical devices such as stents [59] (Fig. 2a) and neural electrodes [40,43] include anti-bacterial [36,37,41,42], anti-coagulation [37], anti-inflammatory activities [37], nutrient delivery to surrounding tissues [40,43], and prevention of implant-related infection in orthopedics [35,39]. Examples of drug-loaded hydrogel coatings are listed in Table 1, including the loaded drugs or nanoparticles, the constituents of the hydrogel coatings and the nature of the bonding between the hydrogel coatings and substrates. In order to load drugs into hydrogel coatings, the drugs are generally dispersed in the hydrogel precursor, and the subsequent curing entraps the drugs inside the hydrogel coating. Another approach is to immerse a cured hydrogel coating in a drug solution. The release rate of the loaded drugs is determined by the diffusion of the drugs into the surrounding tissues and can be accelerated by using a biodegradable polymer network to construct the hydrogel coating. Moreover, by tailoring the molecular interactions between the hydrogel polymer network and the drugs—i.e. covalent linkage, electrostatic interaction and hydrophobic interaction—the drug release rate can be efficiently tuned.

### Lubricity

Some lubricious biological surfaces, such as the cartilage of animal joints, are essentially hydrogels consisting of fibrous collagen and proteoglycans. Synthetic hydrogels, with non-adhesive dangling chains on their surfaces, are able to achieve an extremely low coefficient of friction, on the order of 10^−4^ [60]. The tribological behavior of hydrogels is significantly different to that of solids and is influenced by several factors including the chemical structure of the hydrogel, the surface properties of the sliding surface and the measurement [61]. One explanation attributes the low friction to the presence of a hydrated layer between the hydrogel and the sliding substrate [61]. The lubricity of the hydrogel-coated surface is important for some medical applications, such as contact lenses [10], catheters [45,46,62] and medical guidewire [32]. For example, a catheter with a thin tube structure serves a broad range of functions in treating diseases or performing surgical procedures. Catheters are inserted into a body cavity,

duct or vessel, allowing drainage and administration of fluids or gases and access for other surgical instruments, such as guidewire. The typical biomaterials for the construction of catheters are polymers, such as silicone and poly(vinyl chloride) (PVC), which are inert and unreactive to body fluids but experience high friction with surrounding tissues and are likely to suffer biofouling. Direct insertion of an uncoated catheter is likely to cause trauma to the surrounding tissue during the operation. Lubricious biocompatible hydrogel coatings enable the insertion of catheters into tortuous anatomical pathways with reduced tissue irritation (Fig. 2b) [31]. Another example is metal guidewire, which is a medical device used to deliver implants such as stents to a desired site. Making guidewire surface lubricious is instrumental to easing surgical operation and alleviating patient discomfort (Fig. 2c) [32].

### Anti-biofouling

The term biofouling describes the contamination of surfaces by the adhesion of organisms and their by-products [63]. Surface biofouling of implanted medical devices is caused by the adhesion of microbial or thrombotic agents due to foreign body response [64]. Biofouling limits the lifetime of implanted medical devices, and can even result in their removal and replacement [50]. Biofouling is a complex process related to the physical and chemical properties of the target surface. The anti-biofouling properties of implanted medical devices can be enhanced by surface modifications, including controlling surface hydrophilicity and charge, biomolecule functionalization, and drug elution [64]. For hydrophilic surfaces, resistance to the adhesion of fouling agents is ascribed to the hydration layer formed between the coating and the surrounding environment. This hydration layer serves as a physical barrier to resist the adhesion of fouling agents. Hydrogels, as well-known hydrophilic materials, can substantially enhance the hydrophilicity of the coated surface. Hydrogels commonly used as anti-biofouling coatings on implanted medical devices include poly(vinyl alcohol) (PVA), polyethylene glycol (PEG) and natural polysaccharides, such as chitosan and dextran [47,48,50,52]. PEG has been credited as a gold standard material [64]. The positive charges on the backbone of polysaccharide hydrogels are thought to be effective in disrupting the lipid membrane of microbes, resulting in antimicrobial activity. Another category of anti-biofouling hydrogel coatings is zwitterionic hydrogels, which are neutral but have an equal number of positive and negative charges along the polymer network [49,51]. The most frequently studied zwitterionic materials are prepared by adhering poly(sulfo-betaine) and poly(carboxybetaine) to methacrylate or acrylamide backbones. The hydration layer formed on the surface of the zwitterionic hydrogel coatings is strongly bonded to the coating surface through electrostatic interactions.

### Conductive coatings for neural electrode

Mobile ions obtained by dissolving salt in the water component of hydrogels endow hydrogels with ionic conductivity. The resistivity of salt-containing hydrogel can be reduced to ∼10^1^ Ωm, compared with ∼18.2 MΩm for pure water [19]. Living matter mostly uses ions to conduct electrical signals, while machines exclusively use electrons. An emerging research field, known as hydrogel ionotronics, has been rapidly evolving in recent years [19]. A hydrogel-based ionic cable mimics the function of an axon in terms of ionic conductivity [20]. A hydrogel-based ionic conductor can transmit electrical signals at high speed, 16 orders of magnitude higher than the diffusivity of ions in solution, enabling the ionic conductor to transmit electrical signals with high frequency (up to 100 MHz) over a distance of 10 cm. Furthermore, an ionic conductor can transmit enough power to turn on a light-emitting diode, even if the resistivity of the ionic conductor is several orders of magnitude higher than that of an electronic conductor [20]. In medical applications, hydrogel ionic conductors inherit the biocompatibility of the hydrogel and are ideal conductive coatings for neural electrodes. Neural electrode probes, which mainly consist of rigid metals, such as platinum, gold and iridium for the electrode, and silicon, polyimide and ceramic for the construction material, are electronic medical devices implanted into the brain or other electrically excitable tissue to record electrical signals or stimulate neurons with electrical impulses. Hydrogel coatings serve as a mechanical buffer between a rigid neural electrode and soft tissue, attenuating the formation of glial scars resulting from the trauma induced by the micro-motion between the electrode and tissue [44,55,65,66]. Glial scar encapsulation on the electrode surface increases the impedance between the electrode and tissue, resulting in the failure or degradation of the neural signal transmission. When coated with conductive hydrogel, the chronically implanted neural electrode maintains a low impedance over 1 billion stimulations (Fig. 2d) [58]. The biocompatibility of the hydrogel coating also effectively reduces the loss of the neural cells around the electrode [44]. Furthermore, the conductivity between the electrode and tissue can be enhanced by depositing a layer of conductive polymer—poly(3, 4-ethylenedioxythiophene) (PEDOT)—on the metal electrode before coating with the hydrogel [56,57]. Nerve growth factor-loaded hydrogel coating is able to enhance neuron survival and promote the differentiation of neural cells around the electrode [40,43].

### Sensing

The diversity of hydrogel chemistry enables a wide range of responses, for example deformation and change of transparency, to various stimuli such as temperature, pH, magnetic field and chemicals. Stimuli-responsive hydrogels are appealing candidates for soft actuator and soft sensor applications. Stimuli-responsive hydrogel coatings incorporated with other substrates or devices generate several new sensing systems. Hydrogel interferometry prepared by coating stimuli-responsive hydrogel on silicon wafer is sensitive to volatile vapor, humidity, copper ions and glycoprotein through the change of hydrogel thickness, presenting different visible structure colors due to the interference of the light reflected by the hydrogel and silicon wafer surface (Fig. 2e) [67,68]. As the detection is based on the diffusion of target small molecules to the hydrogel coating through the thickness direction, the thin hydrogel coating (on the order of 100 nm) used in this sensing system enables a fast response to an equilibrium state. The implementation of this sensing system is the result of the synergy of chemistry, mechanics and optics. A hydrogel-coated fiber Bragg grating (FBG) allows the measurement of salinity. Under different saline concentrations, the hydrogel coating swells to reach different equilibrium states, resulting in varied FBG stretching and the corresponding shifts in the wavelength of the reflected ‘Bragg’ signal. In principle, this sensing system can be extended for the detection of other chemical species through modification of the gel chemistry with the same sensor construction [71]. Based on surface plasmon resonance spectroscopy, glucose phosphate barium salt-imprinted hydrogel coating on a gold surface yields a selective sensor for glucose. When gold nanoparticles are incorporated into the hydrogel coating to give a higher refractive index, the sensitivity of the sensor is significantly enhanced, making the sensor capable of detecting glucose at a level of μg/mL in deionized water [70].

### Actuation

The stimuli-responsive hydrogels used as active materials for actuation feature a relatively large actuation deformation triggered by external stimuli, such as pH, temperature, magnetic field and hydraulic or pneumatic pressure. The stimuli responses of hydrogels are readily implemented by using a stimuli-responsive polymer network for hydrogel formation (e.g. poly(*N*-isopropylacrylamide) for temperature response, and polyacrylic acid for pH response); embedding active elements in the hydrogel matrices (e.g. magnetic particles for magnetic field response); and designing structures with chambers or channels for actuation by hydraulic or pneumatic pressure [34]. Active hydrogels for actuation that are in coating form are mostly based on stimuli-responsive polymer networks. Under stimuli, the conformational transformation or change of crosslink density of the polymer network induces swelling/deswelling of the hydrogel, resulting in an expansion or contraction for actuation. A bilayer structure is the most commonly used configuration for the construction of a stimuli-responsive hydrogel coating-based actuator [73,76]. The two layers can be prepared from different stimuli-responsive hydrogel coatings, achieving reversible and bidirectional actuation. Using the bilayer structure as a primary element, complex configuration transformation from 2D to 3D can be achieved via structure design or patterned active hydrogel coating [72,75]. Furthermore, temperature-responsive hydrogel coatings can drive the motion of fluids in microfluidic devices by cyclic swelling and deswelling stimulated by temperature [74]. For more information on hydrogel actuators, please refer to previous reviews [88].

### Anti-marine creature fouling

A submerged surface in the marine environment suffers the accumulation of marine fouling organisms, such as algae, diatoms and barnacles, known as marine creature fouling [89]. Marine creature fouling slows vessels, resulting in additional energy consumption and vessel maintenance costs. Furthermore, the life of the hull can be shortened owing to the corrosion caused by the attached organisms. The fouling process is affected by several physical and chemical properties of the surface, such as the surface tension, wettability, modulus, roughness and chemistry. Coating the target surface with anti-fouling materials is the mostly widely adopted approach to achieving anti-fouling properties for submerged substrates in the marine environment [89]. The earliest coating materials include pitch, tar, wax and heavy metals such as lead. However, these materials have a low durability and limited anti-fouling performance. In the mid-1960s, a more durable tributyltin (TBT)-containing self-polishing anti-fouling coating was formulated, which shows prolonged anti-fouling efficiency for up to five years as a result of the continuous release of TBT from the coating. However, TBT was banned in 2008 owing to its adverse effects on aquatic life [82]. Exploring environmentally friendly, durable anti-marine creature fouling coatings has since been urgently pursued to protect submerged surfaces and the marine environment. Examples of such functional coatings include natural anti-fouling compounds, silicone and fluorocarbon polymers with low surface energy, and hydrogels. The anti-fouling characteristic of hydrogel coatings is attributed to their highly hydrated surfaces. Several functional hydrogel coatings, such as PVA and poly(2-acrylamide-2-methyl-propanesulfonic acid)/polyacrylamide (PAMPS/PAAm) double network tough hydrogel, PEG and zwitterionic hydrogels, have been shown to be effective in inhibiting the attachment of marine creatures in experiments [77–82]. For example, a polyacrylamide hydrogel-coated model boat floating in a water-filled tank contaminated with oil shows anti-oil fouling properties (Fig. 2f) [32]. Functional hydrogel coatings therefore have potential for application where adhesion by marine creatures is undesired.

### Oil-water separation

Oily wastewater pollution is becoming a worldwide threat to marine and aquatic ecosystems owing to increasing oily wastewater from industry and frequent oil spill accidents [86]. The demand for reusable oil-water separation materials with high efficiency and low cost is becoming increasingly urgent. Due to the intrinsic immiscibility of water and oil, materials with extreme affinity either to water or oil are promising candidates for application in oil-water separation [90]. ‘Oil removing’ materials are superhydrophobic or superoleophilic materials that allow the infiltration of oil while leaving the water on the other side [83]. Examples of these materials include polyester textiles, carbon-based materials, hydrophobic aerogels, polystyrene and polytetrafluoroethylene (PTFE) coating mesh. However, these materials are prone to pore clogging and surface fouling by oil due to their intrinsic oleophilic properties, resulting in the degradation of separation efficiency and a short life.

In contrast to ‘oil removing’ materials, ‘water removing’ materials are superhydrophilic and underwater superoleophobic and do not suffer the limitations of ‘oil removing’ materials. Hydrogels are well-known hydrophilic materials whose water component can exceed 95% in weight. Hydrogels themselves as base materials for oil-water separation have the disadvantage of low water flux—below 30 L/m^2^  h^−1^  bar^−1^—as the mesh size is on the order of 10 nm [91]. Coating commercial filtration films with hydrogels combines the advantages of the high flux of the filtration film and the hydrophilicity of the hydrogel [83,84,87]. Gao *et al.* coated a thin alginate hydrogel film onto the surface of a polyacrylic acid-*grafted*-poly(vinylidene fluoride) (PAA-*g*-PVDF) filtration membrane via layer-by-layer self-assembly of sodium alginate and Cu^2+^. This hydrogel coated PAA-*g*-PVDF filtration film achieved a high water flux of up to 1230 L/m^2^ h^−1^ bar^−1^ with high separation efficiency—above 99.8%—and an outstanding cyclic performance [86]. Instead of coating hydrogel on the surface of the filtration membrane, Xue *et al.* coated micro-scale porous metal substrates, with mesh sizes ranging from 34 to 380 μm, with rough nanostructured hydrogel [85]. This hydrogel-coated mesh exhibited selective and effective (>99%) separation of water from various oil/water mixtures including vegetable oil and even crude oil. These superhydrophilic and underwater superoleophobic hydrogel-coated filtration films for oil/water mixture separation have the advantages of non-fouling and cyclic usage, which make them appealing candidates in industrial oily wastewater treatment and oil spill cleanup.

## HYDROGEL COATING METHODS

An ideal hydrogel coating method should achieve two goals: strong adhesion to the substrate and conformation to a substrate with an arbitrary shape. Strong adhesion requires a strong interaction, such as covalent bonding, at the interface of the hydrogel coating and substrate, and strong cohesive strength of the hydrogel coating itself. The strong adhesion prevents the delamination or fracture of the hydrogel coating due to cyclic swelling or repeated sliding against the surroundings, while a method of coating a substrate with an arbitrary shape is important. Uniform hydrogel coatings can easily be cast on a flat substrate with a mold, but coating on a substrate with a complex shape can be challenging. In the following section we only describe the coating methods that meet the above two requirements.

### The surface bridge method

The adhesion of hydrogels to other substrates by simple attaching is intrinsically low owing to the presence of abundant water at the interface [17]. Strong hydrogel adhesion is the result of strong interaction between the polymer network of the hydrogel and the target substrate. The surface bridge method for strong adhesion complies with the following principle: the two ends of a bridge molecule can form a strong interaction with the hydrogel and substrate separately, establishing strong bonding at the substrate-coating interface.

**Figure 3.**
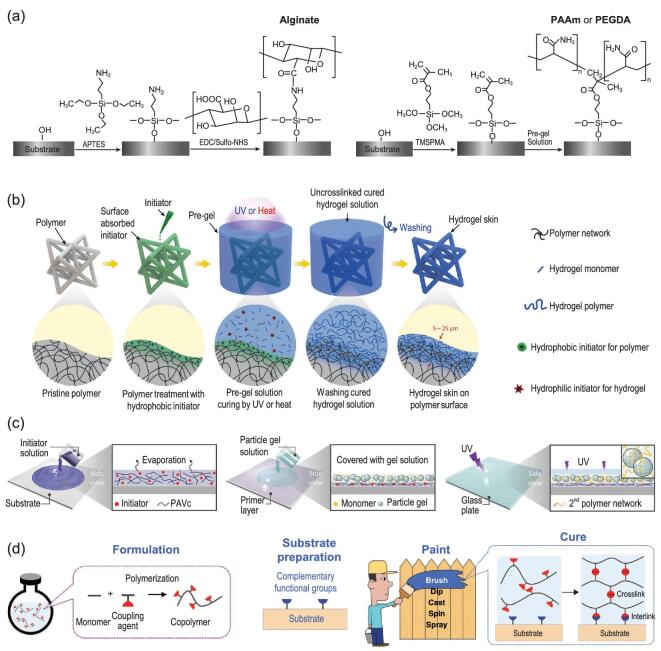
Hydrogel coating methods. (a) The surface bridge method. The bridge molecules used are (3-aminopropyl) triethoxysilane (APTES) and 3-(trimethoxysilyl) propyl methacrylate (TMSPMA). The two ends of the molecules form covalent bonds with the substrate and hydrogel coating separately, enabling strong bonding of the hydrogel coating to the substrate. The figure is redrawn with permission from ref [24], Springer Nature. (b) and (c) The surface initiation method. Hydrophobic benzophenone photo-initiator is adsorbed onto the surface of the target substrate by diffusion or an additional primer. The subsequent curing of a hydrogel precursor on the treated substrate enables a hydrogel coating to be strongly bonded to the substrate. (b) is adapted with permission from ref [31], John Wiley and Sons. (c) is adapted with permission from ref [30], John Wiley and Sons. (d) The hydrogel paint method. A paste-like hydrogel paint is applied to a pre-treated substrate using common painting operations such as brushing and dipping. The crosslinking of the hydrogel coating and strong bonding formation between the coating and substrate complete in the curing process of the hydrogel paint. The figure is adapted with permission from ref [32], John Wiley and Sons.

The commonly used bridge molecules for hydrogel coatings are silanes or silane-coupling agents such as 3-(trimethoxysilyl) propyl methacrylate (TMSPMA) and (3-aminopropyl) triethoxysilane (APTES), as shown in Fig. 3a [24,92]. The alkoxy groups at one end hydrolyze into silanol groups in an aqueous environment and condense with hydroxyl groups on the target surface to form siloxane bonds, enabling strong bonding between the target substrate and the bridge molecules. The other end of the APTES has an amino group that can condense with a carboxyl group on the polymer network of hydrogels such as alginate and hyaluronan by EDC-Sulfo NHS chemistry. For TMSPMA, the vinyl group at the other end can participate in the polymerization of the hydrogel-coating precursor, enabling covalent bonding between the hydrogel coating and the TMSPMA.

A hydrogel coating can be firmly bonded to a target substrate through bridge molecules. This method was first used by Yuk *et al.* for bonding tough hydrogels to non-porous surfaces, such as metal, glass, silicon and ceramics, enabling adhesion of more than 1000 J/m^2^ to be achieved [24]. In principle, as long as the substrate is rich in surface hydroxyl groups the method is valid. Inorganic solids, such as glasses, metals and ceramics naturally have hydroxyl groups on their clean surface exposed to the air. While for some organic solids, such as elastomers and plastics, the hydroxyl groups can be obtained by surface treatments such as oxygen plasma and UV ozone [32].

A hydrogel precursor is usually cast on the treated substrate and then cures in a mold to hold the shape and to isolate oxygen, which prohibits the polymerization of hydrogel precursor [24,25]. In principle, coating hydrogel on a substrate with an arbitrary shape can be readily achieved by the surface bridge method as long as the hydrogel precursor can be grafted onto the substrate without a mold. A printable hydrogel precursor, which behaves like a paste, meets this requirement and can be applied to the target substrate by brushing or dipping. The printable hydrogel precursor can be prepared by adding a rheology modifier, such as nano-clay, micro-gel or long natural or synthetic polymer chains to the water-like hydrogel precursor [93]. To spread the hydrogel precursor on the target substrate steadily, the surface energy of the substrate should be higher than the surface tension of the hydrogel precursor. The surface energy of the target surface can be increased using numerous techniques including plasma treatment, corona treatment and acid etching. Conversely, additives such as surfactants can be added to the hydrogel precursor to reduce its surface tension. The subsequent curing of the hydrogel precursor can be carried out in an oxygen-free environment, usually in a nitrogen-filled chamber. The loss of water during curing is another consideration. Humectants such as lithium chloride can be added to the hydrogel precursor or the chamber humidity can be kept high to alleviate the water loss.

The validity of the surface bridge method in hydrogel coating formation relies on the functional groups in the bridge molecules. For example, the widely used TMSPMA works for substrates with abundant hydroxyl groups on the surface and hydrogels prepared by free-radical polymerization of vinyl monomers. In contrast, APTES is suitable for hydrogels that are rich in carboxyl groups in the polymer network. Furthermore, it is possible to design new bridge molecules and incorporate different functions, such as on-demand breakage. For example, Li *et al.* designed a bridge molecule containing a carboxylic acid group at one end, which realized strong bonding with the metal surface through coordination of deprotonated carboxylate groups to metal ions, and electrostatic and hydrophobic interactions, and a methacrylic group at the other end, which chemically bonded with the hydrogel network through copolymerization. By introducing a breakable disulfide bond in the bridge molecule, they achieved on-demand debonding of the hydrogel–metal interface by stimulation with reduction agents such as glutathione [94].

### The surface initiation method

Benzophenone is a hydrophobic photo-initiator that can diffuse into the surface of polymers such as polydimethylsiloxane (PDMS), polyurethane, latex, VHB (Very High Bond) and Ecoflex with the help of an appropriate organic solvent. The benzophenone absorbed in the elastomer serves as a grafting agent to bond the polymer network of the hydrogel to that of the elastomer and as an oxygen scavenger to alleviate the oxygen inhibition effect [25]. When exposed to ultraviolet (UV) light, a benzophenone molecule abstracts a hydrogen atom from the polymer network of the substrate and generates a free radical, mediating the grafting of the hydrogel network to the polymer network of the elastomer [25]. The validity of the surface initiation method using benzophenone is limited to polymers that can swell in benzophenone organic solution and supply hydrogen to benzophenone for free-radical generation. Two different methods of forming a hydrogel coating on benzophenone-adsorbed polymers have been adopted. The first involves the application of a paste-like hydrogel precursor to the target surface and then exposing it to UV light for curing, as described for the surface bridge method [29]. Owing to the lower surface energy of most polymers compared with that of the hydrogel precursor, which mainly comprises water, additional treatment of the polymer surface to increase its surface energy—for example with oxygen plasma—is necessary to ensure a steady spread of the hydrogel precursor on the polymer. Otherwise, the hydrogel precursor might bead on the hydrophobic polymer surface.

An alternative method is to immerse the treated polymers in a water-like hydrogel precursor, which is a mixture of water, hydrogel monomer and hydrophilic initiator, as shown in Fig. 3b [31]. The hydrophilic initiator initiates the polymerization of hydrogel monomers within and above the surface-bound diffusion layer of the substrate. Meanwhile, the hydrophobic initiator initiates the polymerization inside the diffusion layer of the polymer, grafting the hydrogel polymer chains to the network of the substrate. In addition to the hydrophobic photo-initiator (benzophenone), a hydrophobic thermo-initiator (benzoyl peroxide) has also been used as another example of this method. Thermal initiation is advantageous in cases where reaching the desired hydrogel coating site with the light required to initiate polymerization is challenging [31]. As a result, the polymer networks of the substrate and hydrogel interpenetrate at the interface and form covalent linkages, achieving strong bonding. The weakly attached polymer chains on the surface of the elastomer can be rinsed away with water, leaving a strongly bonded ‘hydrogel skin’ on the target substrate. This method enables a ‘hydrogel skin’ on polymers with an arbitrary shape, a tunable thickness ranging from 5 to 25 μm and resistance to prolonged shear forces.

The surface initiation method was further developed by Takahashi *et al.* to achieve hydrogel coating of substrates that are unable to adsorb benzophenone directly by diffusion [30]. Their method consists of two steps, as shown in Fig. 3c. The target substrate, regardless of whether it is plastic, rubber, ceramic or metal, is coated with a primer, which is a mixture of benzophenone and poly(vinyl acetate) (PVAc). This primer layer physically bonds with the target substrate with strong adhesion. A paste-like hydrogel precursor is then applied to the treated surface, followed by photo-initiated polymerization in an oxygen-free environment. High adhesion of over 1000 J/m^2^ was achieved. The coatings are wear resistant and stable after soaking in pure water in an ambient environment for 282 days.

In principle, the surface initiation method is suitable for hydrogel coating formation on most substrates. For polymers, the hydrophobic initiators can diffuse into their surface with the help of an appropriate solvent (e.g. ethanol, acetone). For other substrates, such as metals and ceramics, an initiator containing primer, such as poly(vinyl acetate), can be applied on the target surface to introduce the initiators. The surfaces of most polymers have low surface energy compared with that of water, which hinders the wetting of the hydrogel precursor on the target substrate. To achieve steady spreading of a paste-like hydrogel precursor on the substrate or better diffusion of the hydrogel monomers into the target surface, a substrate with high surface energy is preferred for better wetting. To meet this requirement, surface treatment (e.g. plasma treatment, corona treatment and acid etching) of the target surface can be carried out for a higher surface energy, or additives (e.g. surfactant) can be added to the hydrogel precursor to lower the surface tension.

### The hydrogel paint method

In general, hydrogel coating formation using free-radical polymerization involves three processes: polymerizing hydrogel monomers to polymer chains, crosslinking the polymer chains to give a hydrogel network, and bonding the network to the target substrate. These processes occur concurrently in one step. For example, a polyacrylamide hydrogel coating on a silica substrate is completed in a one-step polymerization between the vinyl groups of the acrylamide monomers, the crosslinker (N, N^′^-Methylenebisacrylamide, MBAA) and the silane molecules (TMSPMA) anchored on the silica substrate. The disadvantages of this traditional approach are clear: (i) the handling of the toxic hydrogel monomers and initiators is not user-friendly; (ii) free-radical polymerization usually applies to an oxygen-free environment; (iii) a liquid-like hydrogel precursor requires a mold to hold its shape before solidification, which hinders the implementation of hydrogel coatings on complex structures.

Recently, a new method known as the hydrogel paint method has been put forward for coating different substrates with various hydrogels, as delineated in Fig. 3d [32,95]. To make a hydrogel paintable, the key is the decoupling of the three processes of hydrogel coating formation given above. As schematically illustrated in Fig. 3d, the hydrogel paint is a solution of copolymers of hydrogel monomers and coupling agents, and behaves like a viscous liquid or a common paint. The functional groups on the coupling agents have tunable kinetics to interact with each other and with the complementary functional groups on the substrate for hydrogel paint curing and strong bond formation after applying the hydrogel paint to the target substrate using various painting technologies. Several embodiments of hydrogel paint are achieved by copolymerizing various hydrogel monomers, such as acrylamide for lubricious, acrylic acid for pH-sensitive and *N*-isopropylacrylamide for temperature-responsive applications, with silane coupling agent (TMSPMA) through vinyl groups. The condensation rate of silanol groups in hydrolyzed TMSPMA molecules in the copolymers and the hydroxyl groups on the target substrate can be tuned using pH and curing temperature. Accordingly, the shelf life or curing time of hydrogel paint can be tuned from hours to days. Furthermore, the shelf life of the hydrogel paints can be significantly extended to months by freeze drying, storing and re-dissolving in water.

The hydrogel paint strategy enables the division of labor. The paint maker is responsible for paint formation using sophisticated chemistry, while the paint user need not be hampered by the need for sophistication and is responsible for substrate preparation, painting and curing. The toxic monomer and initiator are mostly consumed during paint formation so that the paint user does not need to handle the toxic compounds. The tunable viscosity of hydrogel paint, using either water content or rheology modifiers, allows it to be applied and grafted onto substrates with complex structures. The oxygen insensitivity of hydrogel paint curing and bonding with the substrate, for example through silane condensation, ensures the hydrogel coating formation is successful in an ambient environment. All of these advantages make the hydrogel paint method an appealing way of hydrogel coating formation in real applications.

The functional groups or coupling agents in hydrogel paints determine their applications and validities. For example, silanol groups can condense with hydroxyl groups on target substrates for strong bonding, and condense with each other for solidification of the hydrogel paint. Silane condensation is oxygen insensitive. Thus, the bonding and curing of hydrogel paint can be carried out without precluding oxygen. The hydrogel paint method of hydrogel coating was relatively recently introduced and is open for further exploration and development.

### Purification of hydrogel coatings

Free-radical polymerization of hydrogel precursors, which mainly consist of monomers, crosslinkers and initiators, is a prevailing method in hydrogel preparation. The conversion efficiency of hydrogel monomers to a hydrogel network does not reach 100%, which leaves unreacted monomers and initiators that are toxic and may leach out of the hydrogel matrix. The removal of these residual small molecules before use is important in real applications, particularly in biomedical areas. The common procedure for removing these undesirable small molecules is by incubating the cured hydrogel in excess deionized (DI) water for several hours to days with regular replenishment of the DI water [96–99]. The time for the diffusion of these small molecules out of the hydrogel depends on the characteristic size of the hydrogel sample $L$, which can be estimated as ${{{L^2}} \mathord{/ {\vphantom {{{L^2}} D}} \kern-\nulldelimiterspace} D}$, where $D$ is the diffusion coefficient of these small molecules in DI water (typically on the order of 10^−9^ m^2^/s at room temperature).

## ADHESION OF HYDROGEL COATINGS

Adhesion between the hydrogel coating and substrate, which is the energy needed to peel away a unit area of hydrogel coating and has units of J/m^2^, quantifies the resistance to debonding of the coating. However, rigorous characterization of the adhesion of hydrogel coatings is lacking for three main reasons: first, strong adhesion of hydrogel coatings to substrates is relatively new. Second, hydrogels have only recently been integrated with other materials such as dielectric elastomers in hydrogel ionotronics and metals in biomedical devices. Third, the thickness of the hydrogel coating can be as thin as several microns, which makes testing adhesion challenging. In this section, we discuss the origin of the thickness-dependent adhesion of hydrogel coatings and the test methods used to test the adhesion.

### Thickness-dependent adhesion

**Figure 4.**
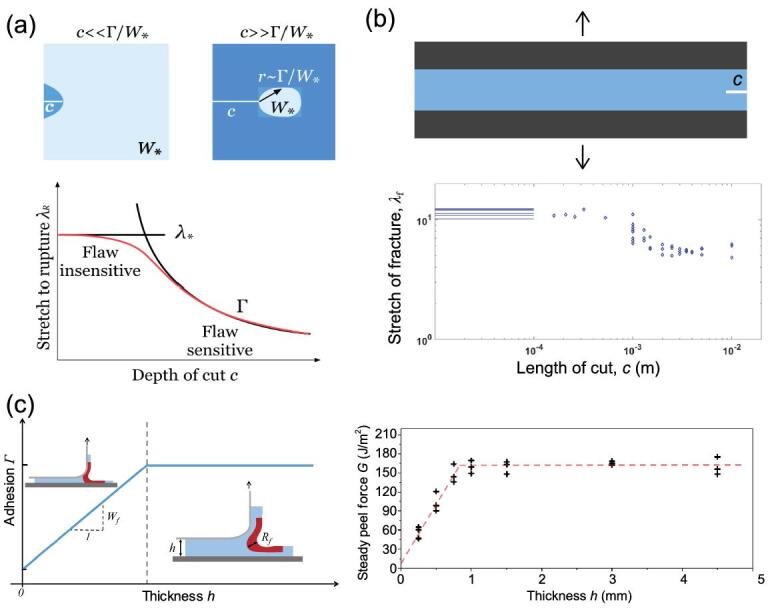
Fractocohesive length of a hydrogel. (a) The fractocohesive length of a hydrogel, estimated by the ratio of ${\Gamma/{{W_f}}}$, determines the transition flaw length of the hydrogel—from flaw insensitive to flaw sensitive—with the existence of a flaw. The figure is adapted with permission from ref [101], Elsevier. (b) The rupture stretch of a piece of polyacrylamide hydrogel is almost a constant with pre-existing flaws or cracks of lengths smaller than the fractocohesive length (around 1 cm in this case). Otherwise, it decreases with increasing flaw size. The figure is redrawn with permission from ref [100], Elsevier. (c) Left: schematic diagrams of the adhesion of a hydrogel coating as a function of coating thickness in a peel test. Right: experimental results for the adhesion of polyacrylamide hydrogel coatings with various coating thicknesses in peel tests. The figure is adapted with permission from ref [102], Elsevier.

A material-specific length, termed the fractocohesive length, was recently defined [100]. This length is the ratio of ${\Gamma \mathord{/ {\vphantom {\Gamma {{W_f}}}} \kern-\nulldelimiterspace} {{W_f}}}$, where $\Gamma $ is the material toughness obtained by stretching a sample with a crack and ${W_f}$ is the fracture work obtained by stretching a sample without a crack. The fractocohesive length is comparable to the flaw sensitivity length of the material. The ultimate strength of the material, such as ultimate stretch, is not affected by the existence of a crack that has a feature size smaller than the flaw sensitivity length (Fig. 4a and b) [100,101]. The fractocohesive length can be compared with the size of the fracture process zone, which is a highly stretched zone in the vicinity of the crack tip. The material in the fracture process zone is highly stretched, dissipating energy through inelastic processes (such as viscoelasticity, poroelasticity for hydrogels and chain scission) and storing elastic energy by elastic deformation. A recent theory indicates that both the energy dissipated during loading and the release of the stored elastic energy corresponding to crack propagation contribute to the toughness as well as the adhesion of the hydrogel coating [102,103].

Peeling away a hydrogel coating involves two length scales: the thickness of the hydrogel coating and the fractocohesive length of the hydrogel. The difference between the two length scales defines two cases: small-scale and large-scale inelasticity peel. If the coating thickness is smaller than the fractocohesive length, the fracture process zone is bounded by the coating thickness. We call this case the large-scale inelasticity peel. In this case, the adhesion increases as the coating thickness increases. Otherwise, the fracture process zone is bounded by the fractocohesive length, which is a constant and independent of the coating thickness (Fig. 4c). The thickness dependent adhesion of viscoelastic adhesive has been well studied both in theory and experiment [104,105]. In light of thickness-dependent adhesion, it is important to clarify the thickness of the hydrogel coating when specifying the adhesion.

Another commonly used length scale is called the elasto-adhesive length, defined as ${\Gamma/E}$, where $E$ is the Young's modulus [106]. This length scale defines the size of a nonlinear elastic region at the crack front where linear elastic fracture mechanics (LEFM) are no longer valid. Considering the high stretchability of soft materials, the elasto-adhesive length can be greater than the fractocohesive length by several orders of magnitude.

### The peel test

**Figure 5.**
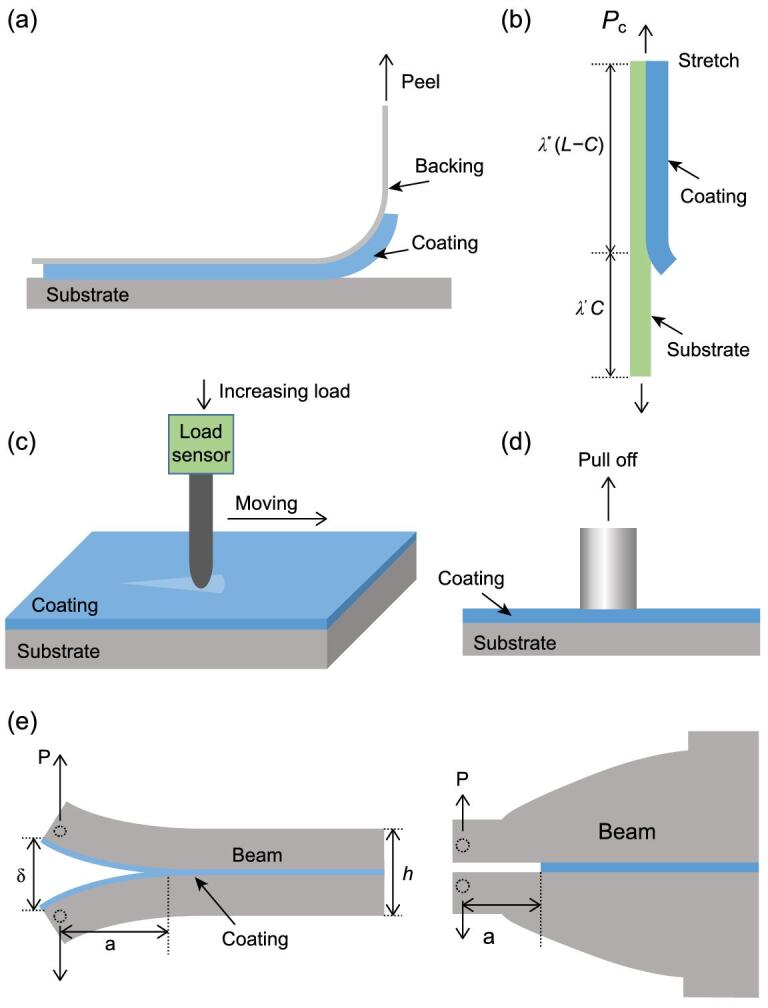
Test methods for the adhesion of hydrogel coatings. (a) The 90 degree peel test. A hydrogel coating is peeled off the substrate by a backing attached at an angle of 90 degrees to the substrate. (b) The simple stretch test. A stretchable substrate coated with hydrogel coating is loaded by uniaxial stretch, resulting in debonding of the coating under a critical load. (c) The scratch test. A rigid stylus is drawn across the surface of the coating, accompanied by a synchronous movement in the thickness direction of the coating. (d) The probe pull test. A flat tipped indenter is bonded to a hydrogel coating and then pulled away. (e) The double cantilever beam test (left) and contoured double cantilever beam test (right). A hydrogel coating is sandwiched between two cantilever beams. The two beams are pulled apart, resulting in fracture of the hydrogel coating.

To test the strength of the adhesion of a hydrogel coating to a target substrate, the coating must be peeled off the substrate either through the substrate/hydrogel interface or through the hydrogel coating. The peel test is typically used to evaluate the adhesion of adhesive tapes [107] and has been further developed to test the adhesion of metal and ceramic films [108,109]. Recently, this method has been adopted to test the adhesion of hydrogel coatings. To peel off a hydrogel coating, a flexible and inextensible backing layer (typically a polyester film with a thickness of around 100 μm) is generally attached to it (Fig. 5a). A peeling result is related to many factors, including the peel speed, the backing layer used, the mode of bonding the backing to the hydrogel coating, and the angle through which the hydrogel coating is peeled off. The interpretation of the peel results is based on the work balance in steady crack propagation, in which the crack propagates at a constant speed while the peel force is invariant over peel distance. Considering the constant elastic energy stored in the hydrogel coating and neglecting the elastic energy stored in the flexible and inextensible backing, the work imparted by the steady peel force is a direct reflection of the adhesion of the hydrogel coating. For simplicity, the backing layer can be pulled at an angle of 90 (Fig. 5a) or 180 degrees to the substrate, giving an adhesion of ${{{F_{ss}}} \mathord{/ {\vphantom {{{F_{ss}}} h}} \kern-\nulldelimiterspace} h}$ or ${{2{F_{ss}}} \mathord{/ {\vphantom {{2{F_{ss}}} h}} \kern-\nulldelimiterspace} h}$, where ${F_{ss}}$ is the steady peel force and *h* is the width of the hydrogel coating. A general expression is $\Gamma = {{{F_{ss}}} \mathord{/ {\vphantom {{{F_{ss}}} {h( {1 - \cos \theta } )}}} \kern-\nulldelimiterspace} {h( {1 - \cos \theta } )}}$, where $\theta $ is the peel angle with respect to the substrate. It is noted that the steady peel force is a function of the peel angle $\theta $ [110]. At different peel angles, the deformation states at the crack front of the hydrogel are different, resulting in different crack modes. Although the peel test gives a global result for adhesion, namely the energy needed to peel off a unit area of hydrogel coating, it reveals nothing about the mechanism of debonding [106].

An experimental challenge of the peel test is how to firmly bond the backing layer to the hydrogel coating without disrupting the coating or debonding during the test. Cyanoacrylates (known as superglues) are commonly used to achieve strong bonding. Cyanoacrylate monomers diffuse into the hydrogel polymer network and form polycyanoacrylate chains through anionic polymerization in topological entanglement with the hydrogel polymer network. Meanwhile, the polycyanoacrylate chains cause densely packed non-covalent interaction with the non-permeable plastic backing [111]. When the hydrogel coating is very thin, the diffusion of the cyanoacrylate into the hydrogel leads to appreciable influence on the integrity of the hydrogel coating, compromising the peel results. The diffusion depth of cyanoacrylate into hydrogel has been found to reach ∼10 μm [112]. The peel test requires a new approach for achieving strong bonding between the coating and the backing layer. Nevertheless, if the thickness of the hydrogel coating is much greater than the diffusion depth of the cyanoacrylates, the side effects induced by the adhesive are negligible.

### The simple stretch test

This method is effective for testing weakly bonded hydrogel coatings on stretchable substrates, for example a weakly bonded hydrogel coating on an elastomer such as PDMS or VHB [113]. When applying stretch to the specimen, as shown in Fig. 5b, the coating and substrate deform and store elastic energy. Assuming the specimen undergoes uniaxial tension, we denote the stretch in the coating/substrate bilayer segment $\lambda ^{\prime\prime}$ and that in the substrate single layer segment $\lambda ^{\prime}$. The combination of the elastic energy stored in the specimen and the potential energy of the applied force give the potential energy of the system as $U = C[ {{U_s}( {\lambda ^{\prime}} ) - P\lambda ^{\prime}} ] + ( {L - C} )[ {{U_b}( {\lambda ^{\prime\prime}} ) - P\lambda ^{\prime\prime}} ]$, where ${U_s}$ is the elastic energy stored in a unit area of the substrate, ${U_b}$ is the elastic energy stored in a unit area of the coating/substrate bilayer, and $P$ is the force per unit width of the hydrogel coating. The applied energy release rate is $G = - {{\partial U} \mathord{/ {\vphantom {{\partial U} {\partial C}}} \kern-\nulldelimiterspace} {\partial C}}$. Accordingly, we get $G = {U_b}( {\lambda ^{\prime\prime}} ) - {U_s}( {\lambda ^{\prime}} ) + P( {\lambda ^{\prime} - \lambda ^{\prime\prime}} )$. Considering that the hydrogel coating is usually thin and has low modulus, the bonded hydrogel coating has negligible influence on the deformation of the substrate, yielding $\lambda ^{\prime\prime} = \lambda ^{\prime}$. The energy release rate is therefore simplified to $G = {U_b}( {\lambda ^{\prime}} ) - {U_s}( {\lambda ^{\prime}} )$, which implies that the release of the elastic energy stored in the hydrogel coating contributes to the crack propagation. If the hydrogel coating is much stiffer than the substrate (for example, one bonds an inextensible backing on the other side of the hydrogel coating), the stretch of the bilayer segment is negligible, thus $\lambda ^{\prime\prime} = 1$. In this case, the energy release rate is $G = - {U_s}( {\lambda ^{\prime}} ) + P( {\lambda ^{\prime} - 1} )$. This energy release rate is ascribed to the release of the elastic energy in the substrate and the change of the potential energy by the applied force. The energy release rate ${G_c}$ corresponding to the propagation of the crack under critical pull force ${P_c}$ gives the adhesion of the hydrogel coating.

### The scratch test

In this test, a rigid stylus is drawn on the surface of the coating, accompanied by a synchronous movement in the thickness direction of the coating in a continuous or stepwise manner, as shown in Fig. 5c. The force on the stylus is recorded during scratching and the critical force at which the failure of the coating occurs is determined [114]. The scratch test is regarded as semi-quantitative in determining the adhesion of the coating because the critical scratch force extracted from the test is affected by numerous factors that are not adhesion-related, such as scratching speed, stylus tip radius and substrate hardness, among others. Interpreting the critical scratch force in terms of the adhesion using a mechanical model is intractable [115]. Efforts have been made for limited cases, such as hard coating/soft substrate systems, and for limited failure modes, such as coating detachment ahead of the stylus [116,117]. Careful theoretical considerations or simulations are required to relate the experimental results to the adhesion of the coating. Fortunately, the scratch test has the advantages of being easy to use and no special specimen preparation being required. The critical scratch force obtained is effective as quantitative but relative data to evaluate adhesion between coatings [118].

### The probe pull test

In the probe pull test, a cylindrical, flat or hemispherical-ended probe is bonded to the hydrogel coating. The probe then pulls the coating off the substrate with a constant velocity, as schematically shown in Fig. 5d [119]. The pull force as a function of pull distance is recorded, which is mainly characterized by four parameters: the peak stress ${\sigma _{\max }}$, the maximum extension ${\varepsilon _{\max }}$, the plateau stress ${\sigma _p}$ and the work of debonding ${W_{{\rm{deb}}}}$. Like the adhesion obtained in the peel test, the four parameters obtained in the probe pull test are quantitative evaluations of different hydrogel coatings under various test conditions. The probe pull test has the same issue with bonding the probe and the hydrogel coating together as the peel test. If the probe debonds along the interface with the coating during the test, the pull result fails to reflect the bonding between the hydrogel coating and the target substrate.

### The double cantilever beam test

In this test, a coating is bonded between two cantilever beams, as illustrated in Fig. 5e [120]. A load is then applied vertically to the end of the beam. The load is denoted $P$ and the deflection between the two ends of the beams is denoted $\delta $. The coating between the beams has a pre-crack of length $a$. Based on a simple beam model, in which the length of the beam equals the crack length $a$ and the boundary of the beam at the crack tip is fixed, the energy release rate is given by ${G_I} = \frac{{3B{P^2}{a^2}}}{{2b}}$, with $b$ being the width of the specimen and $B = \frac{{64}}{{Eb{h^3}}}$ being a constant of the beam ($E$ is the Young's modulus of the beam, and ${h \mathord{/ {\vphantom {h 2}} \kern-\nulldelimiterspace} 2}$ is the thickness of each beam). If the modulus of the coating is much lower than that of the two bonded beams, the soft coating will give rise to a deflection of the beams in the uncracked zone. The simple beam theory is not sufficient for this case. Instead, the energy release rate $G$ was derived from a model of a beam on an elastic foundation [121–123]. The shortcoming of the traditional double cantilever beam test is the requirement for accurate measurement of the crack length. A modified double cantilever beam test, known as the contoured double cantilever beam test, was subsequently devised as shown in Fig. 5e [124,125]. If the critical energy release rate is irrelevant to the crack length $a$, one requirement is that the value of ${{B{a^2}} \mathord{/ {\vphantom {{B{a^2}} b}} \kern-\nulldelimiterspace} b}$ is a constant. Thus, the height of the cantilever beams $h$ satisfies ${{{a^2}} \mathord{/ {\vphantom {{{a^2}} {{h^3}}}} \kern-\nulldelimiterspace} {{h^3}}} = {\rm{constant}}$, which gives a contoured profile of the beam. The energy release rate of the coating is obtained once the critical load for crack propagation is measured.

## CONCLUSIONS AND OUTLOOK

In this paper, we review the recent advances in functional hydrogel coatings, with focus on coating functions and applications, coating methods, and coating tests. The majority of the established applications of functional hydrogels are in the field of biomedicine. Hydrogel coating acts as a versatile solution for improving the biocompatibility of preformed materials or devices that will interact with living tissues, such as implants for plastic surgery, artificial menisci, implanted neural electrodes and biosensors. Furthermore, drug-loaded and anti-bacterial hydrogel coatings can reduce the risk of infection and inflammation of living tissues. Functional hydrogel coatings also show huge potential in non-medical applications, such as environmentally friendly anti-fouling coatings for marine vessels, lubricious coatings for soft devices and ionic conductors in stretchable ionotronics. Functional hydrogel coatings are expected to play a key role in various applications.

Hydrogel coating methods have started to achieve tough adhesion in laboratories, but some gaps remain in the translation to mass production. The requirement for surface bridge and initiation methods, such as treatment of the target substrate for strong bonding and an oxygen-free chamber with high humidity for hydrogel coating curing, are accepted in research but may not be economical for mass production in many applications. In addition, the surface initiation method leads to substantial wastage of the hydrogel precursor. The hydrogel paint method uses traditional painting techniques for coating. The paint user is only responsible for the painting and waiting for curing and is free of any involvement with the preparation of the hydrogel paint. However, the current hydrogel paint strategy is only suitable for functional hydrogel coatings with single network and is unable to achieve a very tough hydrogel coating. The compatibility of the hydrogel paint strategy with tough hydrogel formulation approaches such as double networks, and nanoparticle and fiber reinforced composites, has not been investigated so far. The development of tough hydrogel paint requires immediate action.

The strong adhesion between hydrogel coatings and target substrates is the main mechanical consideration in evaluating the quality of functional hydrogel coatings. The incorporation of strong interaction at the coating-substrate interface and the significant dissipation of energy in the bulk of the coating or substrate would give an adhesion of more than 1000 J/m^2^ under static loading conditions. However, the adhesion under cyclic loading conditions has been reported in only a few cases [126–128]. It has been shown that the toughness of a piece of hydrogel under cyclic loading can be two orders of magnitude lower than that under static loading conditions [129]. It is therefore questionable whether the adhesion of the hydrogel coating under cyclic loading is the same as that under static loading. This question is open to substantial further investigation. Several mechanisms have been put forward to improve the fatigue threshold of hydrogels [130–132], and attention should be paid to preparing fatigue-resistant hydrogel coatings.

The long-term stable adhesion of functional hydrogel coatings in harsh environments—such as in seawater and in biological conditions including body fluids and blood—is important for their application. However, studies in this field are limited and scattered. Both the interfacial bonding between hydrogel coatings and substrates and the functional hydrogel coating itself might degrade after extended soaking in these environments, resulting in the failure of the coating by delamination or fracture. For example, a lightly stretched siloxane bond adopted in achieving strong adhesion of a hydrogel coating will hydrolyze into two silanol groups in the aqueous environment [133]. The design of hydrogel coatings that require long-term survival should therefore be carefully considered.

A versatile test method for measuring the adhesion of hydrogel coatings is lacking, particularly when the coatings are extremely thin. The essential difficulty is how to peel the coating off in a quantitative manner. For example, in a peel test, a hydrogel coating is peeled away from a substrate uniformly, assisted by a backing, and the steady peel force is a direct measurement of the adhesion. The peel test is an ideal method for obtaining the adhesion owing to its simple preparation and interpretation, provided that the attachment survives throughout the peel test and its adherence to the coating induces negligible influence on the integrity of the coating. Cyanoacrylate adhesive (commonly used to bond a PET backing to a hydrogel coating) might not be suitable for thin hydrogel coatings owing to the diffusion of the adhesive into the hydrogel. A thin hydrogel coating requires an adhesive-free backing that can bond strongly to the hydrogel coating by attaching, like an adhesive tape. A possible method is to use dense physical bonds, such as hydrogen bonds, to achieve adhesion of a backing to the hydrogel coating. A flexible backing adhered to an acid hydrogel through hydrogen bonds was demonstrated by growing polyacrylic acid (PAA) chains on an elastomer film [134]. The bonding was instant and tough and is attributed to the fast formation of dense hydrogen bonds. The enormous diversity of noncovalent bonds makes the design space for hydrogel adhesive tape applicable to various hydrogels. The development of hydrogel adhesive tapes that are valid for use with various hydrogels and compatible with the peel test is a promising direction for establishing a versatile testing platform for hydrogel coating adhesion. Using this platform, the adhesion test can be completed through a simple attaching and peeling procedure without concerns about the diffusion of additional adhesive into the hydrogel coatings. Because of the size effect in peeling hydrogel coatings and the intrinsic viscoelastic response of polymeric networks, the adhesion of functional hydrogel coatings is specific to the thickness and loading speed. We suggest that the coating thickness and loading speed are made clear when specifying the coating adhesion.
